# Supplementary material for: Cell reprogramming shapes the mitochondrial DNA landscape
Source: Nat Commun. 2021 Sep 2;12:5241. doi: 10.1038/s41467-021-25482-x (PMC8413449; doi:10.1038/s41467-021-25482-x)
Supplement: Supplementary file 1 — Supplementary Information [file 41467_2021_25482_MOESM1_ESM.pdf]

Supplementary Information

Cell reprogramming shapes the mitochondrial DNA landscape

Wei Wei, Daniel J. Gaffney, Patrick F. Chinnery

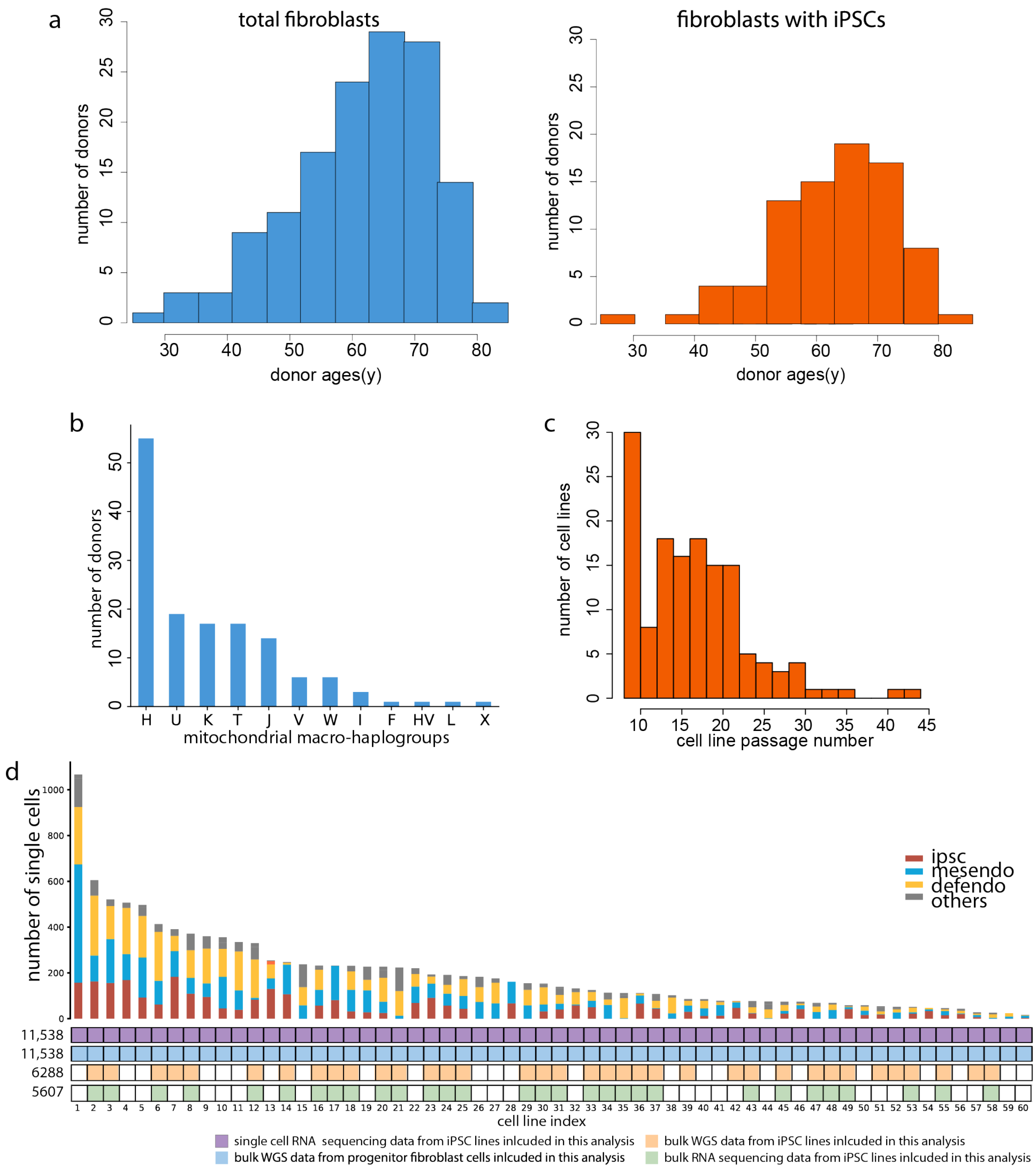
a
total fibroblasts
number of donors
donor ages(y)
fibroblasts with iPSCs
number of donors
donor ages(y)
b
number of donors
H U K T J V W I F HV L X
mitochondrial macro-haplogroups
c
number of cell lines
cell line passage number
d
number of single cells
ipsc
mesendo
defendo
others
11,538
11,538
6288
5607
cell line index
single cell RNA sequencing data from iPSC lines inlcuded in this analysis
bulk WGS data from progenitor fibroblast cells inlcuded in this analysis
bulk WGS data from iPSC lines inlcuded in this analysis
bulk RNA sequencing data from iPSC lines inlcuded in this analysis

Supplementary Fig. 1 Summary of the cell lines included in this study. a Age distribution of the 146 donors contributing fibroblasts (left) and age distribution of the 83 donors contributing iPSCs (right). **b** mtDNA macro-haplogroups defined in the 146 donors. **c** Distribution of iPSC line passage number**. d** Top: the number of single cells from 60 donors analysed in this study. Each bar represents each donor, and cells from three cell stages iPSC, mesendo, defendo and undefined shown in different colours. Bottom: the types of sequencing data available in this analysis. Each square represents each cell line. The numbers of cells sequenced are shown on the left side.

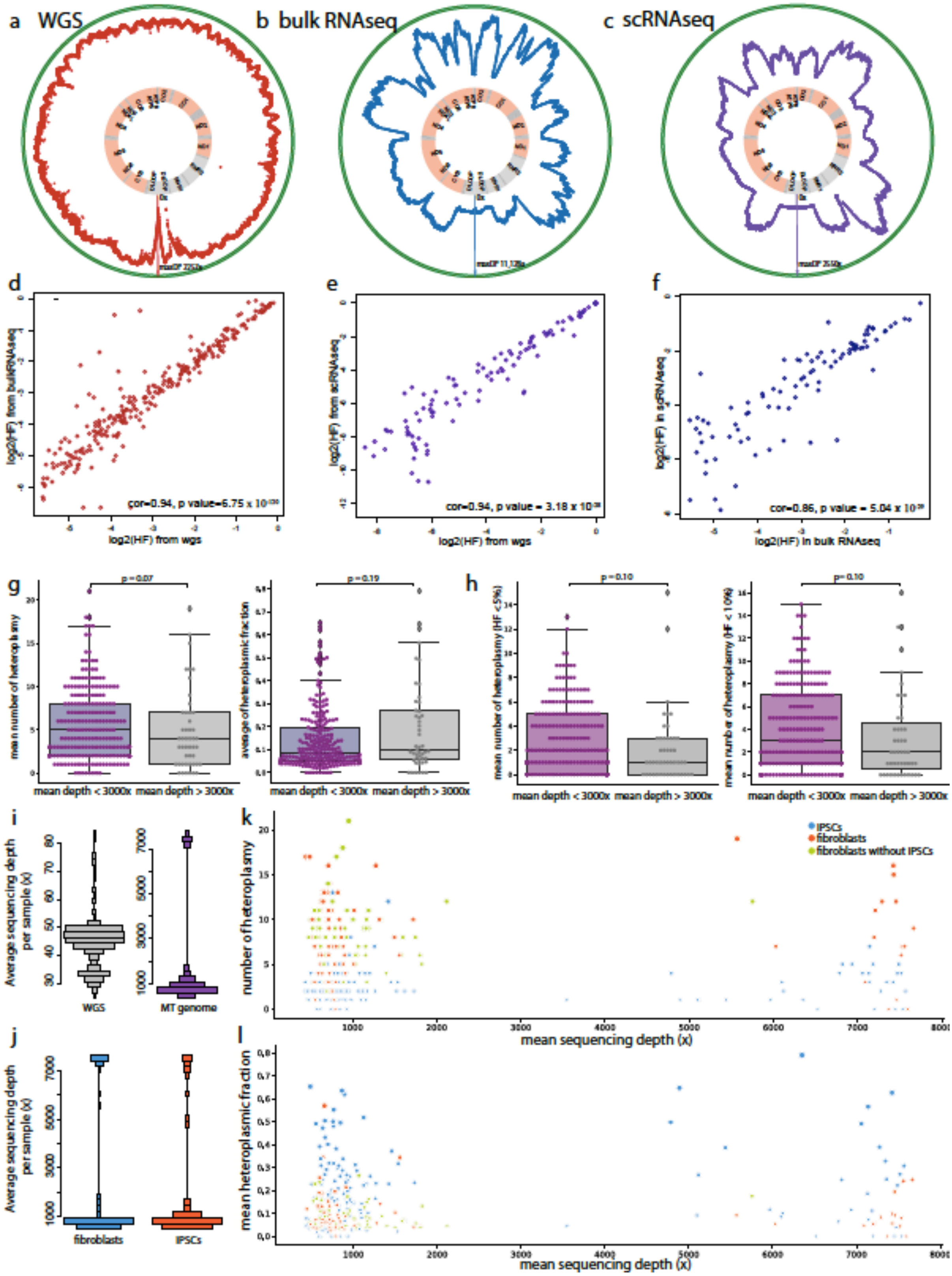

a WGS
b bulk RNAseq
c scRNAseq
d
cor=0.94, p value=6.75 x 10^-130
log2(HF) from wgs
log2(HF) from bulkRNAseq
e
cor=0.94, p value = 3.18 x 10^-38
log2(HF) from wgs
log2(HF) from scRNAseq
f
cor=0.86, p value = 5.04 x 10^-29
log2(HF) in bulk RNAseq
log2(HF) in scRNAseq
g
p = 0.07
mean number of heteroplasmy
p = 0.19
average of heteroplasmic fraction
mean depth < 3000x
mean depth > 3000x
h
p = 0.10
mean number of heteroplasmy (HF < 5%)
mean number of heteroplasmy (HF < 10%)
i
Average sequencing depth per sample (X)
WGS
MT genome
k
number of heteroplasmy
IPSCs
fibroblasts
fibroblasts without IPSCs
mean sequencing depth (X)
j
Average sequencing depth per sample (X)
fibroblasts
IPSCs
l
mean heteroplasmic fraction
mean sequencing depth (X)

Supplementary Fig. 2 Coverage and depth of sequencing. a-c Average sequencing depth of the mitochondrial genome using different sequencing techniques. Inner circle, mitochondrial genome and regions; middle outline, average sequencing depth. From inner to outer, the arrows show the sequencing depth (DP) increasing. The max depth is labelled at the end of each arrow. **d-f** Scatter plot of log2(HF) in heteroplasmic variants from the same iPSC detected between WGS and bulk RNAseq (d), WGS and scRNAseq (e) and bulk RNAseq and scRNAseq (f). P values were calculated using Pearson's correlation test. Source data are provided as a Source Data file**. g** Combined box and swarm plots showing the mean number of heteroplasmic variants and average heteroplasmy fraction for cell lines sequenced at a low or high-depth. The middle "box" represents the median, lower and upper quartile of the data. The upper and lower whiskers represent the data outside the middle 50%. Each dot represents each mitochondrial genome. n = 287 mtDNA sequences. P values were calculated using two-sided Wilcoxon rank sum test. **h** Combined box and swarm plots showing the mean number of low level heteroplasmic variants (left plot HF<5% and right plot HF <10%) for cell lines sequenced at a low or high-depth. The middle "box" represents the median, lower and upper quartile of the data. The upper and lower whiskers represent the data outside the middle 50%. Each dot represents each mitochondrial genome. n = 287 mtDNA sequences. P values were calculated using two-sided Wilcoxon rank sum test. **i** Left: Distribution of the mean whole-genome sequencing depth per sample. Right: Distribution of the mean mtDNA sequencing depth per sample (extracted from the whole genome). **j** Distribution of the mean mtDNA sequencing depth per fibroblast cell line (left) and iPSC line (right). **k** Correlation between the mean sequencing depth of the mtDNA genome with the mean number of heteroplasmic variants. **l** Correlation between the mean sequencing depth of the mtDNA genome and the average heteroplasmy fractions.

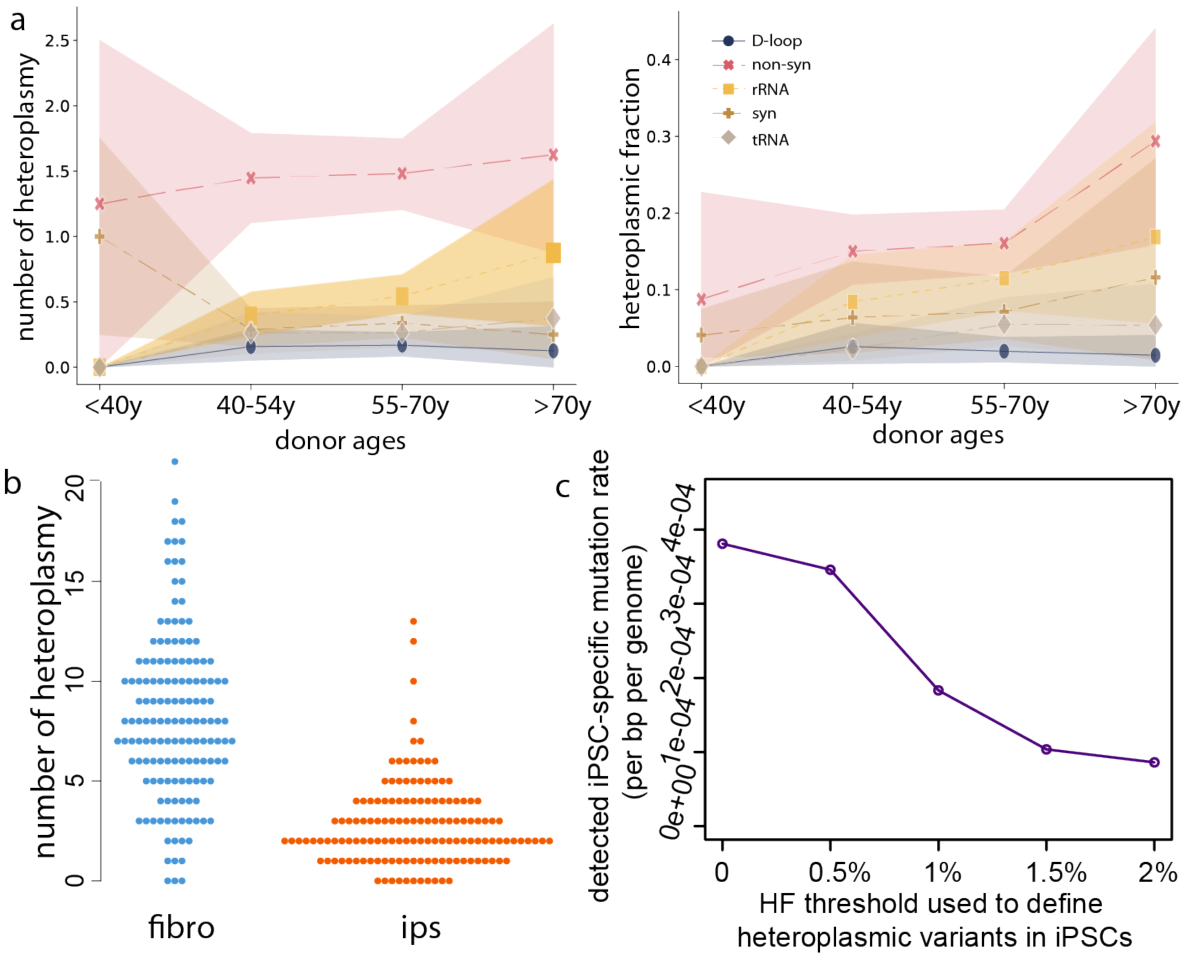

Supplementary Fig. 3 mtDNA variants detected by WGS. a Left: correlation between the mean number of heteroplasmic variants per iPSC line in each mtDNA region with the donors' age. Shaded regions show mean ± standard deviation. Right: correlation between the average of HF per iPSC line in each mtDNA region with the donors' age. Shaded regions show mean ± standard deviation. **b** Swarm plot shows the number of heteroplasmic variants detected in each fibroblast and iPSC cell line. n = 287 mtDNA sequences. Source data are provided as a Source Data file**. c** Detected iPSC-specific mutation rate (per base per genome) using different HF detection thresholds in the iPSC lines.

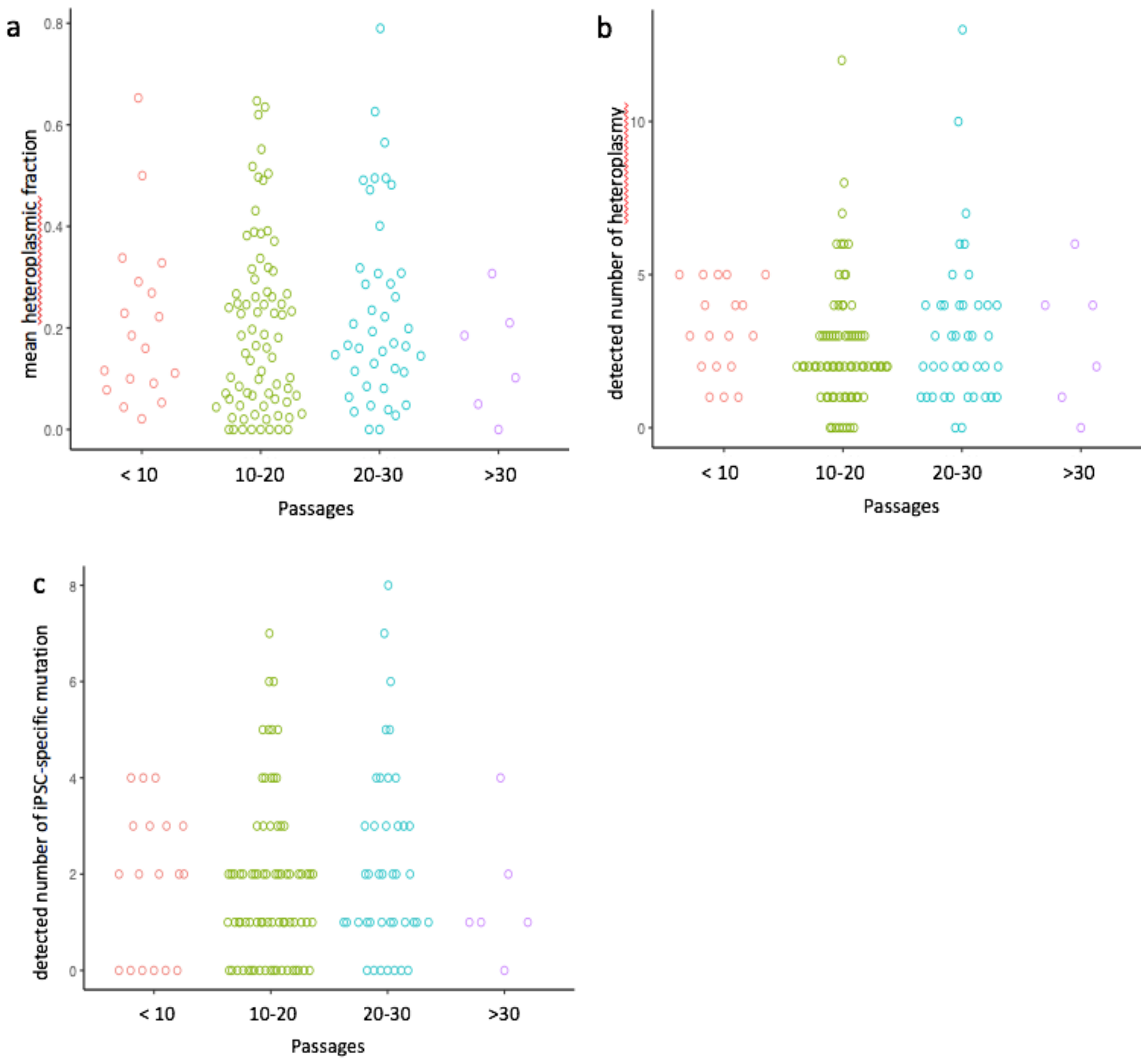

Supplementary Fig. 4 iPSC passages. a Mean heteroplasmic fraction in each iPSC passage group. n = 141 mtDNA sequences. **b** Detected mean number of heteroplasmic variant per iPSC line in each iPSC passage group. n = 141 mtDNA sequences. **c** Detected number of iPSC-specific mutation per iPSC line in each iPSC passage group. n = 141 mtDNA sequences.

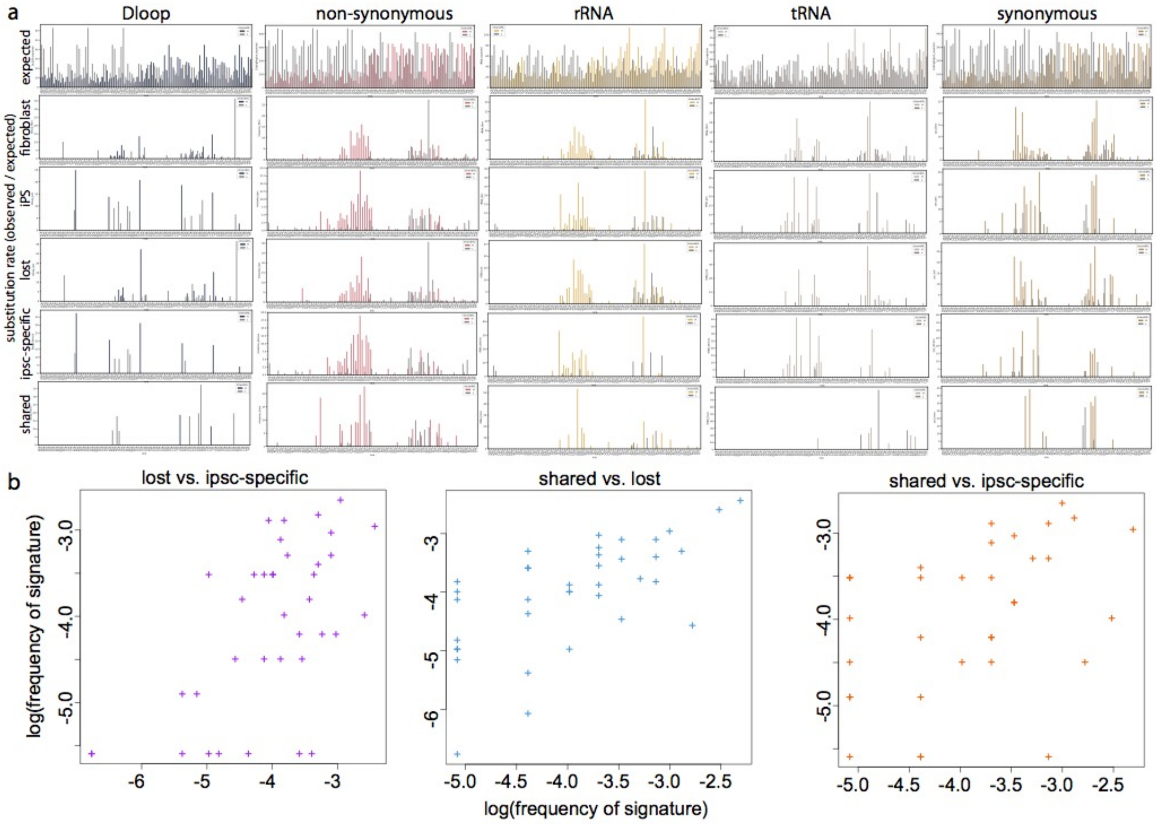

Supplementary Fig. 5 Trinucleotide mutational signature of heteroplasmic variants. **a** Trinucleotide mutational signature of heteroplasmic variants in different mtDNA regions. The substitution rate of expected mutations, variants observed in fibroblasts / iPSCs, lost variants in fibroblasts, iPSC-specific variants in iPSCs, and shared variants between fibroblasts and iPSCs are shown separately. Mutations from the H- or L-strand are shown in different colours. **b** Scatter plot of the log(frequency of mutational signature) between lost and iPSC-specific heteroplasmic variants (left), shared and lost heteroplasmic variants (middle), and shared and iPSC-specific heteroplasmic variants (right).

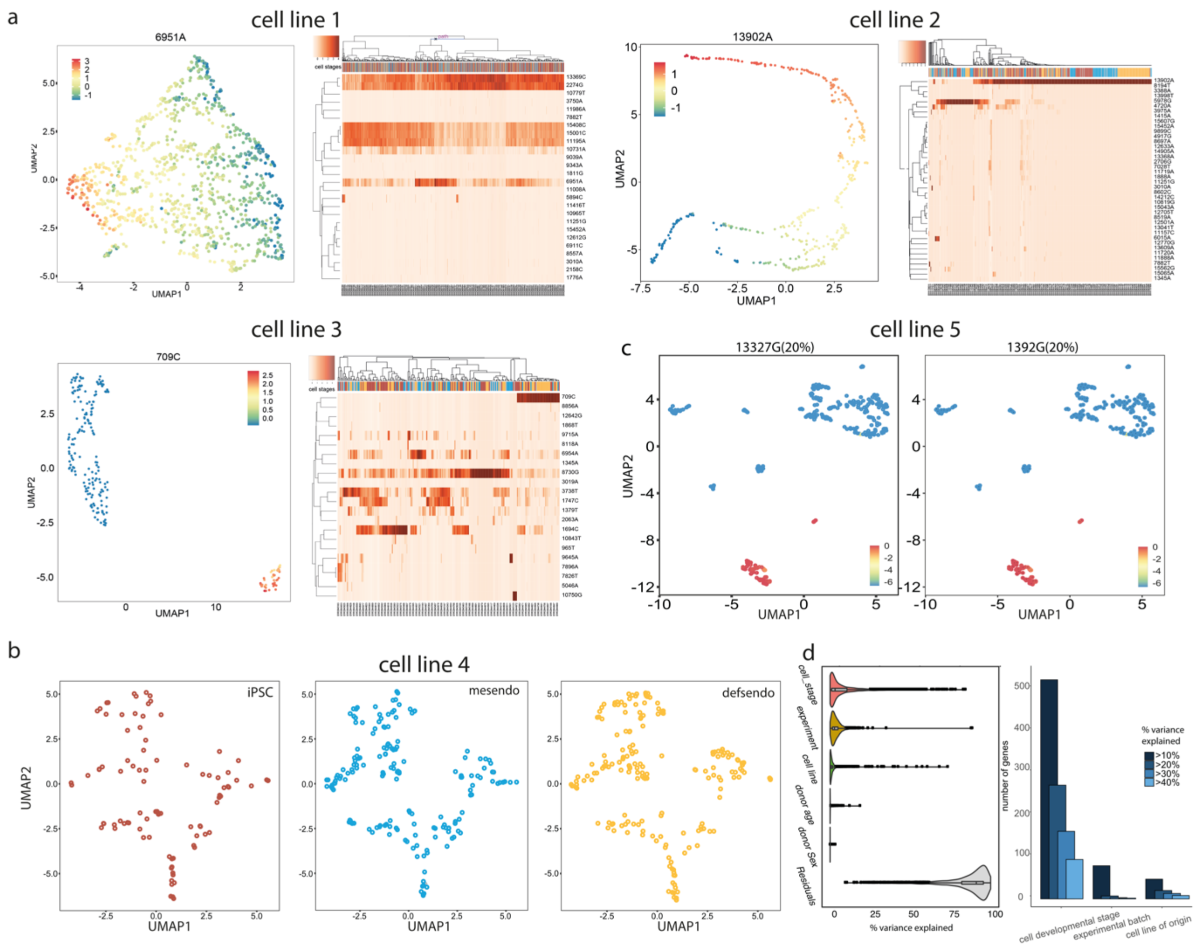
a
cell line 1
6951A
UMAP1
UMAP2
cell stages
cell line 2
13902A
UMAP1
UMAP2
cell line 3
709C
UMAP1
UMAP2
cell line 5
c
13327G(20%)
1392G(20%)
UMAP1
UMAP2
b
cell line 4
iPSC
mesendo
defsendo
UMAP1
UMAP2
d
cell_stage
experiment
cell line
donor age
donor Sex
Residuals
% variance explained
number of genes
% variance explained
>10%
>20%
>30%
>40%
cell developmental stage
experimental batch
cell line of origin

Supplementary Fig. 6 a-c UMAP plots and hierarchical clustering of mtDNA mutation profiles from independent cell lines from single cell RNA sequencing. a UMAP plots of mtDNA mutation profiles with cells coloured by the heteroplasmy fraction of a specific mutation observed in a cell line. The mutations are labelled at the top of the UMAP plots. Hierarchical clustering of mtDNA genotyping (rows) for the single cells – coloured by their cell stages (columns). Colour bar, heteroplasmy fraction. **b** UMAP plots of mtDNA mutation profiles with cells from the same cell line coloured by cell stages. Three cell stages show the similar cluster profile. **c** 13327G and 1392G variants occurring in the same lineage were seen in the same 20% of the cells. **d** Left: Violin and box plots show the percentage of the variance for gene expression explained by cell stage, experiment, cell line, donor age and donor sex. Right: Bar plots show the number of gene with >10% >20% >30% and >40% variance explained by cell stage, experiment and cell line.
