# Supplementary material for: Cell reprogramming shapes the mitochondrial DNA landscape
Source: Nat Commun. 2021 Sep 2;12:5241. doi: 10.1038/s41467-021-25482-x (PMC8413449; doi:10.1038/s41467-021-25482-x)
Supplement: Supplementary file 3 — Reporting Summary [file 41467_2021_25482_MOESM3_ESM.pdf]

nature research

Corresponding author(s): Patrick Chinnery, NCOMMS-21-04418

Last updated by author(s): Jul 25, 2021

Reporting Summary

Nature Research wishes to improve the reproducibility of the work that we publish. This form provides structure for consistency and transparency in reporting. For further information on Nature Research policies, see our Editorial Policies and the Editorial Policy Checklist.

Statistics

For all statistical analyses, confirm that the following items are present in the figure legend, table legend, main text, or Methods section.

n/a	Confirmed	
☐	☒	The exact sample size (*n*) for each experimental group/condition, given as a discrete number and unit of measurement
☐	☒	A statement on whether measurements were taken from distinct samples or whether the same sample was measured repeatedly
☐	☒	The statistical test(s) used AND whether they are one- or two-sided<br>*Only common tests should be described solely by name; describe more complex techniques in the Methods section.*
☐	☒	A description of all covariates tested
☐	☒	A description of any assumptions or corrections, such as tests of normality and adjustment for multiple comparisons
☐	☒	A full description of the statistical parameters including central tendency (e.g. means) or other basic estimates (e.g. regression coefficient) AND variation (e.g. standard deviation) or associated estimates of uncertainty (e.g. confidence intervals)
☐	☒	For null hypothesis testing, the test statistic (e.g. *F*, *t*, *r*) with confidence intervals, effect sizes, degrees of freedom and *P* value noted<br>*Give P values as exact values whenever suitable.*
☒	☐	For Bayesian analysis, information on the choice of priors and Markov chain Monte Carlo settings
☒	☐	For hierarchical and complex designs, identification of the appropriate level for tests and full reporting of outcomes
☐	☒	Estimates of effect sizes (e.g. Cohen's *d*, Pearson's *r*), indicating how they were calculated

Our web collection on statistics for biologists contains articles on many of the points above.

Software and code

Policy information about availability of computer code

Data collection	Not applicable
Data analysis	Public software: MToolBox (v1.1), VarScan2, Samtools (v1.12), Circos (v0.69), Enrichr, UMAP, R (bioconductor) and Python.<br>Custom code is available at: https://github.com/WeiWei060512/HipSci_mtDNA_paper.<br>The details of software and code used in this study are cited and described in Methods.

For manuscripts utilizing custom algorithms or software that are central to the research but not yet described in published literature, software must be made available to editors and reviewers. We strongly encourage code deposition in a community repository (e.g. GitHub). See the Nature Research guidelines for submitting code & software for further information.

Data

Policy information about availability of data

All manuscripts must include a data availability statement. This statement should provide the following information, where applicable:

- Accession codes, unique identifiers, or web links for publicly available datasets
- A list of figures that have associated raw data
- A description of any restrictions on data availability

All HipSci data used in this study can be accessed through http://www.hipsci.org. Whole genome sequencing data is available under accession numbers: ERP017015 (ENA project). Bulk RNA-seq data are available under accession numbers: ERP007111 (ENA project) and EGAS00001001137, EGAS00001000593 (EGA projects). Single cell RNA-seq data are available under the accession numbers ERP016000 (ENA project) and EGAS00001002278, EGAD00001005741(EGA project: study ID, dataset ID). Processed single cell count data are downloaded from Zenodo: https://zenodo.org/record/3625024#.Xil-0y2cZ0s.

Field-specific reporting

Please select the one below that is the best fit for your research. If you are not sure, read the appropriate sections before making your selection.

☒ Life sciences ☐ Behavioural & social sciences ☐ Ecological, evolutionary & environmental sciences

For a reference copy of the document with all sections, see nature.com/documents/nr-reporting-summary-flat.pdf

Life sciences study design

All studies must disclose on these points even when the disclosure is negative.

Sample size	We collected whole genome sequencing data available in HipSci project at the time we performed the analysis, including 151 fibroblast lines and 146 iPSC lines.
Data exclusions	Sequencing data were excluded based on quality control criteria. Potential DNA cross-contamination was checked using mtDNA variant calls. 2 samples carrying more than 10 heteroplasmic variants with similar HFs were excluded from this study. We also removed 4 sequences where the average depth of mtDNA was below 400x. A further 4 iPSCs were removed because to their matched fibroblasts were unavailable or failed quality controls. After all of the sample QC steps, 146 fibroblasts and 141 iPSCs were included in the analysis. The list of cell line IDs is provided below (cell line sources).
Replication	No replication used in this study.
Randomization	iPSC lines were allocated to the experimental batches at random.
Blinding	Investigators were blinded during collection.

Reporting for specific materials, systems and methods

We require information from authors about some types of materials, experimental systems and methods used in many studies. Here, indicate whether each material, system or method listed is relevant to your study. If you are not sure if a list item applies to your research, read the appropriate section before selecting a response.

Materials & experimental systems

n/a	Involved in the study	
☒	☐	Antibodies
☐	☒	Eukaryotic cell lines
☒	☐	Palaeontology and archaeology
☒	☐	Animals and other organisms
☒	☐	Human research participants
☒	☐	Clinical data
☒	☐	Dual use research of concern

Methods

n/a	Involved in the study	
☒	☐	ChIP-seq
☒	☐	Flow cytometry
☒	☐	MRI-based neuroimaging

Eukaryotic cell lines

Policy information about cell lines

Cell line source(s)	All fibroblast and iPSC lines from the HipSci project (www.hipsci.org).<br>Cell line IDs: aion, aion_2, aion_3, babk, babz, babz_3, bezi, bezi_1, bilx, bilx_1, bilx_2, bima, bima_1, bipt, bokz, bokz_5, bokz_6, bubh, burb, burb_1, burb_3, ceik, ceik_1, ceik_2, ciwj, ciwj_1, ciwj_2, cuhk, cups, datg, deyz, deyz_2, deyz_3, diku, doao, doao_1, doao_2, dons, eesb, eesb_1, eika, eipl, eipl_1, eiwy, eiwy_1, eofe, euts, euts_1, euts_3, fafq, fafq_1, fawm, fawm_2, fawm_4, fiaj, fiaj_1, fiaj_3, fiau, fiau_1, fikt, garx, garx_2, gesg, gesg_2, gifk, gifk_1, hajc, hajc_1, hayt, hayt_1, hecn, hecn_3, hecn_6, hehd, heja, heth, heth_1, hiaf, hipn, hipn_1, hipn_2, hoik, hoik_1, ieki, iisa, iisa_3, iiyk, iiyk_2, iiyk_4, iudw, iudw_1, iudw_4, jejf, jejf_2, jejf_3, jilk, jilk_3, jilk_4, jogf, joxm, joxm_1, jufd, juuy, juuy_2, kajh, kajh_2, kajh_3, kefb, kefb_1, kegd, kegd_2, kehc, keui, keui_1, keui_4, kolf, kolf_2, kuco, kuco_1, kuco_5, kute, kute_4, kute_5, kuxp, laey, laey_4, laey_6, lako, lako_1, lako_2, lepk, lepk_1, lepk_4, letw, letw_1, letw_5, lexy, liqa, liqa_1, liqa_6, lise, lise_3, melw, melw_1, melw_2, meue, meue_4, meue_5, miaj, miaj_4, miaj_6, mita, naah, naah_2, naah_4, naju, naju_1, nekd, nufh, nufh_3, nufh_4, nusw, oaaz, oapg, oaqd, oaqd_2, oaqd_3, oevr, oicx, oicx_2, oicx_6, oikd, oikd_2, oikd_5, oilg, oilg_1, oilg_3, paab, paab_3, paab_4, pahc, pahc_4, paim, paim_1, paim_3, pamv, pelm, pipw, pipw_4, pipw_5, podx, puie, puie_4, puie_5, qaqx, qaqx_1, qayj, qayj_3, qayj_4, qehq, qehq_3, qehq_7, qolg, qonc, qoog, qoog_4, qoog_6, quls, quls_2, rayr, rayr_1, riun, riun_1, rozh, rutc, rutc_2, sebn, sebn_3, sebn_4, sebz, sebz_1, sehl, sehl_6, seru, seru_1, seru_7, sita, sita_1, sohd, sohd_2, sohd_3, sojd, sojd_3, suop, suop_2, suop_5, tixi, tixi_4, toco, toco_5, tolg, tolg_4, tolg_6, toss, toss_3, tuju, tuju_1, tuju_4, ualf, ualf_2, ualf_6, uilk, uilk_2, uilk_3, uofv, uolo, uolo_5, vabj, vaka, vaka_5, vass, vass_1, vazt, vazt_1, vazt_2, voce, voce_1, voce_2, vuna, vuna_3, wahn, wahn_1, wahn_2, wetu, wibj, wigw, wigw_2, wiii, wiii_2, wiii_3, wopl, wopl_1, wuye, wuye_2, xojn, xojn_3, xomm, xomm_1, xomm_2, xugn, xuja, yelp, yelp_3, yelp_4, yemz, yemz_1, yoch, yoch_6, zaie, zaie_1, zaie_5, zapk, zerv, zerv_7, zerv_8, zihe, zihe_1, ziyn, ziyn_5, ziyn_6, zoxy
Authentication	The cell lines were not authenticated in this study.
Mycoplasma contamination	All cell lines tested negative for mycoplasma contamination.
Commonly misidentified lines<br>(See ICLAC register)	No commonly misidentified cell lines were used in the study.
