# Supplementary material for: Cell reprogramming shapes the mitochondrial DNA landscape
Source: Nat Commun. 2021 Sep 2;12:5241. doi: 10.1038/s41467-021-25482-x (PMC8413449; doi:10.1038/s41467-021-25482-x)
Supplement: Supplementary file 4 — Description of Additional Supplementary Files [file 41467_2021_25482_MOESM4_ESM.pdf]

Description of Additional Supplementary Files

Supplementary Data 1 Summary of the fibroblast cell and iPS cell lines used in this study.

Supplementary Data 2 Comparison of heteroplasmic shifts in mtDNA regions.

Supplementary Data 3 List of genes with >5% variance explained by mtDNA variants.

Supplementary Data 4 List of differentially expressed (DE) genes (False Discovery Rate, FDR < 10%) detected by mtDNA variants.

Supplementary Data 5 List of Gene Ontology (GO) terms based on DE genes.
